# Exotica: A Further Miscellany of Clinical and Pathological Experiences

**Published:** 1984-10

**Authors:** J. D. Davies

**Affiliations:** Department of Pathology University of Bristol


### Book Review

**EXOTICA: A FURTHER MISCELLANY OF CLINICAL AND PATHOLOGICAL EXPERIENCES**
**W. St. C. Symmers**

**Oxford University Press, Oxford, New York and Toronto, 1984**
**226 pp. +xvii. ISBN 0 19 261225 5. Price £8.95**

Although it does not seem so at the time, 'flu may have its compensations. A recent bout led to a little craftiness. Walking home late one morning in the rain (yes, the car too was under the weather), and all too willing to creep into bed, I knew that there was a copy of Symmers' *Exotica* in my brief case. Even our Secretary and Chief Technician had said please go home. Who would argue in the circumstances?

To those who do not know, William St. Clair Symmers held the chair of Pathology at Charing Cross Hospital Medical School until the year before last. He has been responsible for the production of the most comprehensive of all texts on Systemic Pathology in the English language (6 volumes, 2nd Edition, published by Churchill Livingstone, between 1966 and 1980, if you care to dip into it when not 'flu ridden).

Now I should explain that the main clause of the last sentence is a careful understatement. *Systemic Pathology* is not just a comprehensive reference tome. It is truly English, and its style uses an underrated form of internal dialogue: that is a conversation between the main text and footnotes. Most users of the book are often too busy in acquiring information for examinations or surgical reporting to notice its stylist eccentricities. However I venture to suggest that the book, and its preceding 1st Edition, are to pathology texts what Fielding was to the novel.

*Exotica* has to greater degrees all the hallmarks of individuality. Although published by the august Oxford University Press, the entire book is in the author's own typescript. The frontispiece contains an advertisement poster for a previously ill-attended Lecture Course for students. The modified poster subsequently nearly led to a disciplinary investigation.

The book is divided into 25 short stories which are disguised as medical Case Histories. Just as an appetiser let me tell the constituents of one story, turned into Editorial Key Words: Pigmented fungi and their whims; Misdiagnosis of TB; Cadging colleagues; Guido Banti; Museum pot of 1871.

Such a list might be turned into a medical examination question. Given the above, write an essay to include all the above five topics. 40 minutes, no consultation. See how you get on . . . and then turn up Symmers, Chapter 6, entitled 'Museum piece'.

We all know only too well the measly way in which written medical communication usually takes place. Summaries, Referral and Discharge letters are usually no better than conventional formal papers or chapters. Symmers' book puts all that sort of thing in its proper place. The story behind each tale pokes out: usually amusing or irreverent but always instructive. The contents are relevant to any Consulting Room, Surgery, DGH and even to the most exulted Academic Unit.

The pattern for this book was established in an earlier companion volume, *Curiosa*, which was published in 1974 by Bailliere Tindall, London. So gripping was this predecessor to *Exotica* that it led to embarrassment with a Belgian medical friend who was staying in our house. He is a bon viveur and natural raconteur, well used to the small hours of the morning, but having picked up the book he retired unnaturally early one evening. At the time his hosts were puzzled. It was only in the morning with his wife's complaints that she had not slept for his laughter, that we discovered why.

Now I am not suggesting that you, Indulgent if Suspicious Reader, need any loss of natural sleep. You could ration yourself to one story a night. Alternatively, why not take the book away on a winter holiday? It would be more healthy than any foreign drink taken late at night that I know of. Alternatively, you might think of having a dose of 'flu.

J. D. Davies
Department of Pathology
University of Bristol

